# Intraoperative Discovery of an Unintentionally Retained Abdominal Drain Fragment Incorporated Into the Small Bowel During Elective Colostomy Reversal: A Case Report

**DOI:** 10.7759/cureus.113118

**Published:** 2026-07-21

**Authors:** Unmay S Agarwal, Aniket Khadatkar

**Affiliations:** 1 Department of General Surgery, N.K.P. Salve Institute of Medical Sciences and Lata Mangeshkar Hospital, Nagpur, IND

**Keywords:** abdominal drain kit (adk), colostomy reversal, foreign body incorporation, ileal perforation, intraoperative finding, jejunal erosion, patient safety, retained drain fragment, retained surgical items, surgical complications

## Abstract

Retained surgical items are extremely rare but significant critical complications of abdominal surgery. These complications are usually associated with considerable morbidity and medicolegal implications. Among these, retained surgical drains are especially uncommon and often underreported. We present a case of an incidental intraoperative discovery of a retained abdominal drain fragment during elective colostomy reversal. Here, we present the case of a 40-year-old male who underwent elective reversal of a sigmoid loop colostomy, which had been created 10 months earlier following a penetrating abdominal stab injury. The patient remained asymptomatic during the interim period, with no clinical or radiological suspicion of intra-abdominal pathology. During surgery, dense adhesions were encountered, and a retained fragment of an abdominal drain kit was identified embedded within a fibrous tract. Further exploration revealed a 2 × 1 cm ileal perforation, which had been effectively sealed by the incorporated drain segment, preventing peritoneal contamination. The drain had also eroded proximally into the jejunum and distally into the anterior abdominal wall. The foreign body was carefully removed, the perforation was repaired, and the patient had an uneventful postoperative recovery. Unlike previously reported cases of retained surgical drains, which typically present with infection, obstruction, or fistula within weeks to months of retention, this case is notable for a 10-month asymptomatic interval, attributable to the drain fragment’s dual role in both causing and simultaneously sealing the bowel perforation. This case highlights an unusual presentation in which a retained drain fragment appeared to function as a biological seal over the perforation site, likely accounting for the prolonged asymptomatic course.

## Introduction

Colostomy is a standard operative procedure employed in the management of penetrating abdominal trauma, including stab injuries, to achieve fecal diversion and facilitate distal bowel healing [1]. Colostomy reversal is typically planned after the primary pathology has resolved, clearance of intra-abdominal sepsis, and optimization of the patient’s general condition [2]. Although colostomy reversal is generally considered a safe procedure, it is associated with several potential complications, including adhesion-related complications, anastomotic leakage, and inadvertent visceral injury. Less commonly, reversal may unmask consequences of previous surgery, including the rare but clinically significant problem of a retained surgical item (RSI) [3].

Retained surgical drains represent a distinct and under-recognized subset of RSIs, the broader complication being associated with considerable postoperative morbidity, including adhesion formation, chronic infection, abscess, fistulation, and bowel perforation [3]. While retained sponges and instruments are the most widely documented RSIs, drains, particularly the “abdominal drain kit” (ADK), a disposable closed-drainage system consisting of a soft, radio-opaque drainage catheter connected to a collection bag, commonly used for postoperative abdominal drainage, remain exceedingly rare and comparatively underreported.

While drain-specific incidence data remain limited, RSIs overall occur in approximately 0.3-1.0 per 1,000 abdominal operations, with contributing factors including emergency surgery, unanticipated intraoperative events, prolonged operative duration, and failure to adhere to standardized item-counting protocols [4,5]. Notably, unlike sponges and instruments, drains are not routinely subject to formal counting protocols, which may contribute to their under-recognition when retained.

A systematic review by Indja et al. identified 39 cases of retained surgical drains across abdominal, thoracic, and orthopedic procedures, with drain fracture or transection during attempted removal identified as the most frequent precipitating mechanism, a finding directly mirrored in the present case, in which the retained fragment showed clear evidence of transection at one end, consistent with inadvertent division rather than complete withdrawal. Reported presentations in this literature include chronic pain, abscess formation, fistula development, and migration into adjacent viscera, with most cases ultimately requiring surgical or interventional retrieval [6].

Reporting such cases is essential not only to document unusual intraoperative findings but to reinforce vigilance in surgical practice and strengthen patient safety protocols, in particular, the meticulous documentation of drain type, placement site, and confirmed intact retrieval, the omission of which may allow a retained fragment to go unrecognized. Here, we report the intraoperative discovery of a retained ADK drain encountered unexpectedly during elective colostomy reversal following a stab injury.

## Case presentation

A 40-year-old male resident of Nagpur, India, was admitted to the Department of General Surgery for elective reversal of a sigmoid loop colostomy created following emergency surgery for penetrating abdominal trauma approximately 10 months prior.

Past surgical history

The index surgery had been performed at an outside institution. Operative records from that admission were available and reviewed preoperatively; however, they did not document any complication at the time of drain removal or confirm that drain integrity had been verified. The patient did not return to that institution for follow-up. He had sustained a penetrating abdominal stab injury and was found on emergency exploratory laparotomy to have a through-and-through jejunal perforation with an associated mesenteric tear, both addressed intraoperatively, with a sigmoid loop colostomy fashioned for fecal diversion in view of the degree of contamination. Pelvic and subhepatic drains were placed at closure, brought out through the left and right abdominal walls, respectively. The postoperative course was reported as uneventful, and the patient was discharged in stable condition.

Present admission

Approximately 10 months after the initial surgery, the patient presented for elective colostomy reversal. At the time of admission, the patient was clinically stable and entirely asymptomatic, with no complaints of abdominal pain, fever, nausea, altered bowel habits, or constitutional symptoms. There was no significant medical comorbidity, and the patient denied alcohol consumption, tobacco use, or other substance abuse.

On examination, the patient was afebrile and hemodynamically stable, with a pulse rate of 96 beats per minute and blood pressure of 110/80 mmHg. Systemic examination was unremarkable; cardiovascular and respiratory findings were within normal limits. Abdominal examination revealed a soft, mildly distended abdomen without guarding or rigidity, with a functioning colostomy in situ and audible bowel sounds in all quadrants. Per rectal examination demonstrated normal sphincter tone with no evidence of anorectal pathology or active bleeding.

Investigations

Preoperative radiological assessment confirmed surgical fitness and distal bowel integrity. Chest radiography (posteroanterior view) was unremarkable. Ultrasonography of the abdomen and pelvis was completely normal, with no free fluid, collection, or other finding that would have contraindicated surgery. A Loopogram (a contrast study performed via instillation of water-soluble contrast through the limbs of the stoma, followed by radiographic imaging to assess the de-functioned distal bowel) demonstrated unobstructed contrast passage through both proximal and distal limbs of the colostomy without evidence of stricture, obstruction, or anastomotic leak, thereby confirming distal bowel patency and suitability for reversal.

Operative findings

**Figure 1:**
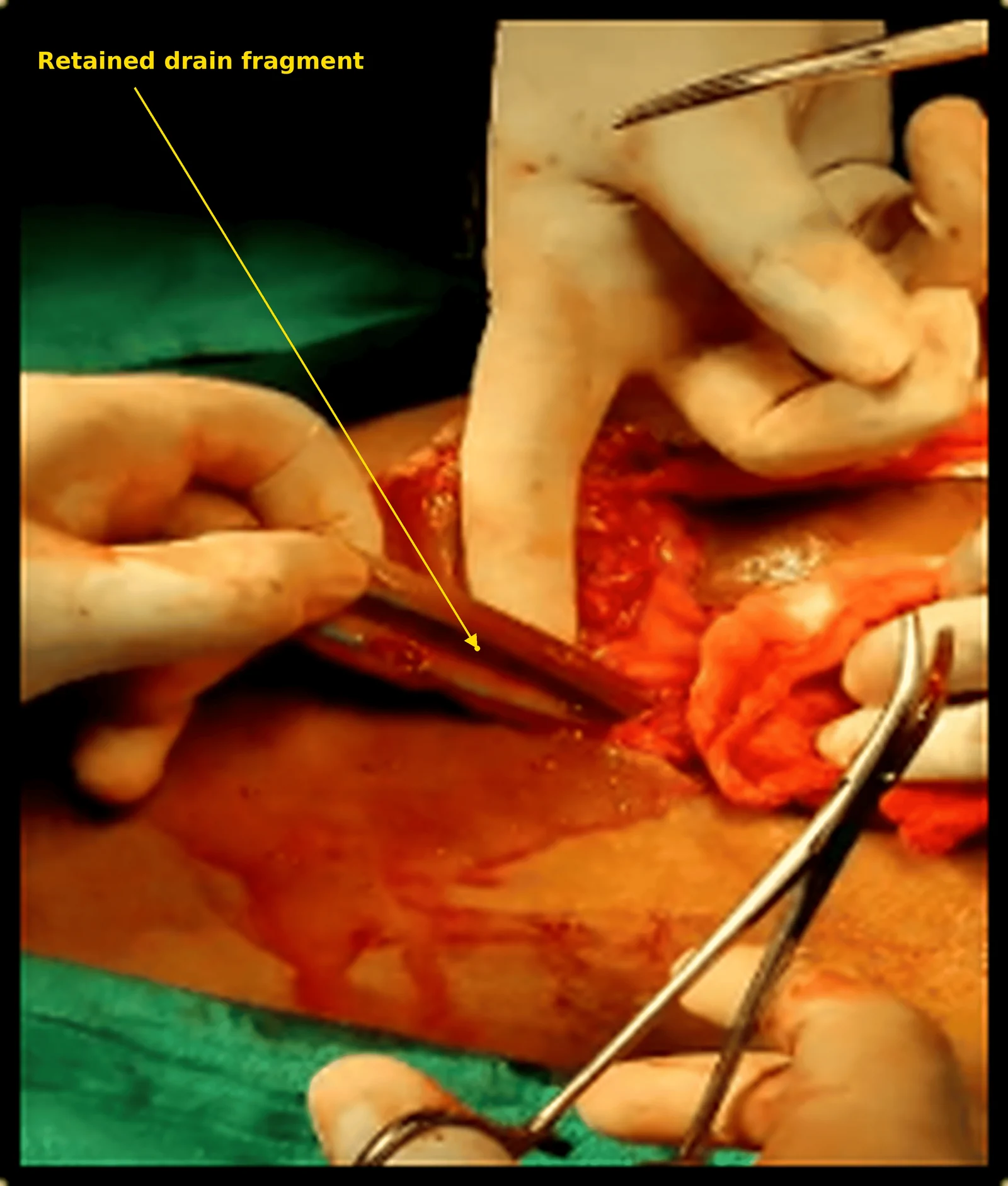
Intraoperative photograph demonstrating the retained drain fragment incorporated within the small bowel, identified during elective colostomy reversal.

Following adequate preoperative optimization, the patient was taken for an exploratory laparotomy and colostomy reversal under general anesthesia. On entering the peritoneal cavity, dense adhesions were encountered in the left pelvic region. Within this region, an unexpected foreign body was identified on careful dissection: a retained ADK drain, partially encased within a mature fibrous tract. The drain was methodically dissected free from surrounding adhesions and retrieved. Examination of the retrieved specimen revealed it to represent only a fragment of the original drain, with evidence of transection at one end consistent with incomplete removal during a prior drain extraction attempt, in which the drain had been divided rather than withdrawn intact (Figure 1).

**Figure 2:**
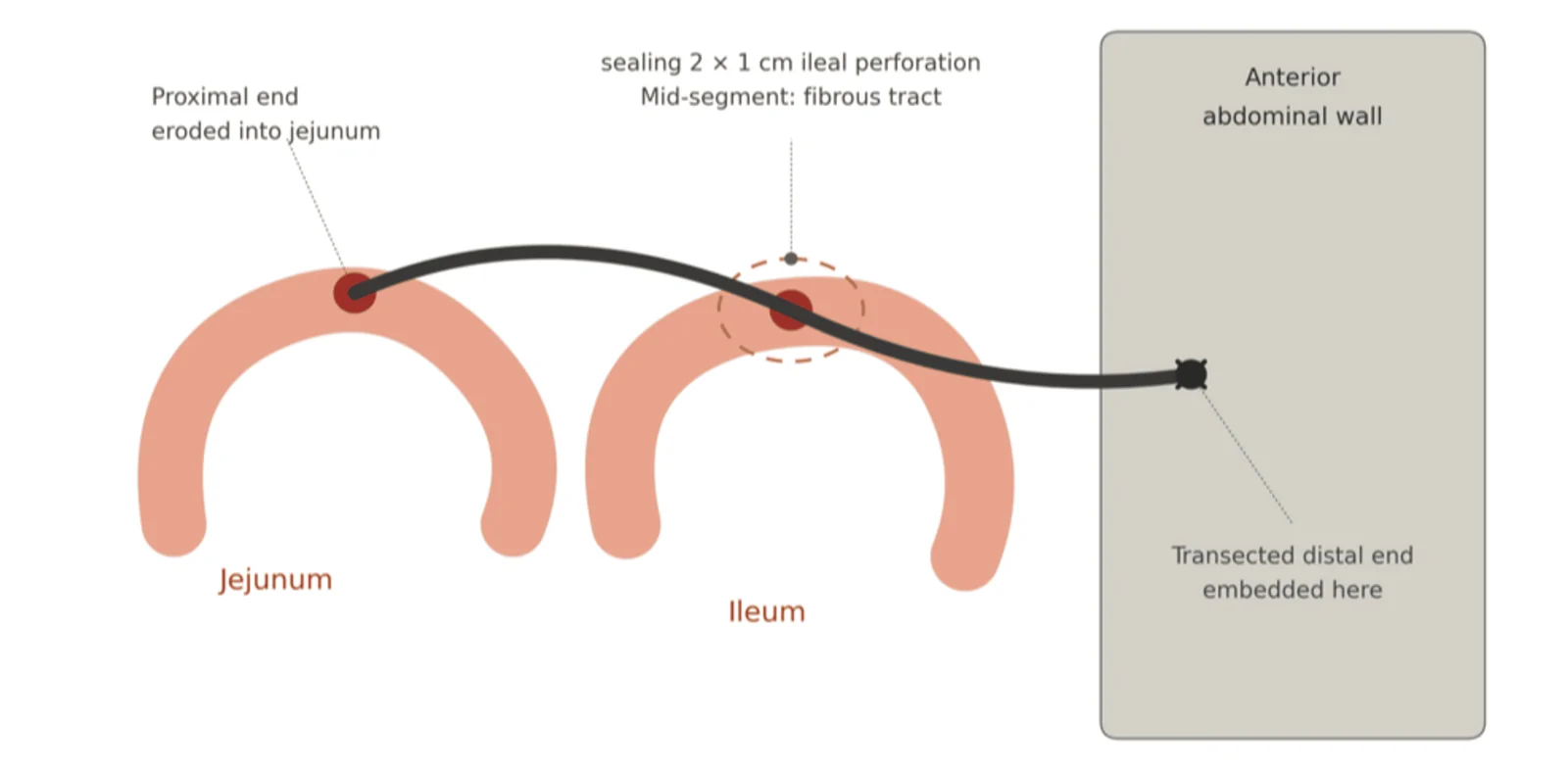
Schematic representation of the retained drain fragment and its relationship to the involved structures. A single fragment courses from its proximal end, eroded into the jejunum, across its mid-segment, which formed a fibrous tract sealing the 2 × 1 cm ileal perforation, to its transected distal end, embedded within the anterior abdominal wall. The transected end corresponds to the site of incomplete removal during the prior drain extraction attempt. The diagram depicts the relational course of the fragment and is not drawn to anatomical scale or orientation.

Systematic intraoperative exploration subsequently revealed a 2 × 1 cm perforation in the proximal ileum. This ileal perforation was anatomically and mechanistically distinct from the jejunal injury sustained and repaired at the index surgery, indicating a new defect resulting from chronic pressure erosion by the retained drain rather than dehiscence or persistence of the original repair. The site of the prior jejunal repair was not specifically visualized during the reversal. The retained drain had bridged this defect, its mid-segment forming a sealed fibrous tract over the perforation site, effectively preventing spillage of intestinal contents and peritoneal contamination. Proximally, the drain had eroded into, but not through, the jejunal wall; this site did not require repair. Distally, the remaining segment was firmly embedded within the anterior abdominal wall and was simply freed without further debridement or excision of the tract (Figure 2). Despite the extent of these findings, there was no intraoperative evidence of peritonitis or free intra-abdominal contamination. Bowel viability at the ileal perforation margins was assessed on visual inspection and tissue color; as the defect involved less than half the bowel circumference with viable margins, primary repair was performed using interrupted 2-0 polyglactin 910 (Vicryl) sutures in a single layer. A leak test performed following repair was negative, and the procedure was completed without further complication.

Postoperative management

**Figure 3:**
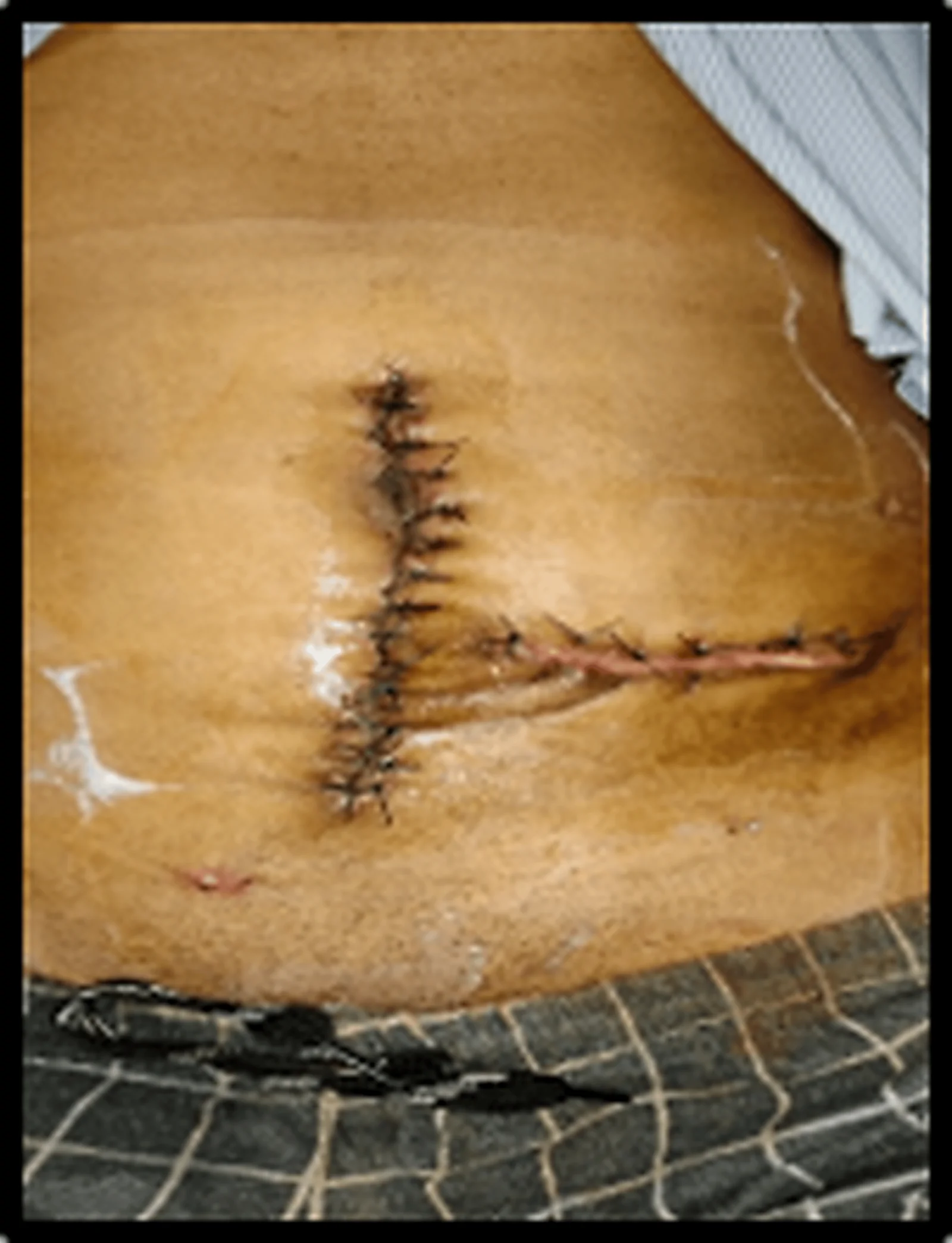
Postoperative photograph showing the closed midline laparotomy incision and the closed previous colostomy stoma site.

Following repair of the ileal perforation and retrieval of the retained drain, thorough peritoneal lavage was performed. A new ADK drain was placed in the right pelvic region, and the abdominal wall was closed in layers (Figure 3). The postoperative course was uneventful; the newly placed drain was removed before discharge, and the patient recovered satisfactorily. The patient was subsequently lost to follow-up, precluding long-term outcome assessment.

## Discussion

Although this case falls within the broader category of RSIs, it differs mechanistically from the classic scenario in which an item is inadvertently left in situ at the index operation. The drain itself had been intentionally and appropriately placed as part of standard postoperative care following the index laparotomy; retention occurred only at the level of a fragment, resulting from incomplete withdrawal during a subsequent removal attempt in which the drain was transected rather than removed intact. This distinction is clinically relevant, as it shifts the focus from item-accounting failures at the time of surgery, the mechanism underlying most reported RSIs, toward failures in the technique and verification of drain removal in the postoperative period.

Although RSIs overall are well documented, with sponges and gauze accounting for approximately 80% of reported cases [5], retained drains are considerably rarer and less systematically characterized. Unrecognized retention of any RSI, regardless of type, may present with chronic infection or abscess, as illustrated by a recent case of gossypiboma presenting as bilateral abdominal abscesses following cesarean section [7].

Two reported cases are particularly instructive in contextualizing the present report. Irpatgire et al. described a corrugated rubber drain retained within the abdominal cavity for five years following exploratory laparotomy, in which repeated ultrasonography failed to identify the drain and diagnosis was ultimately established on CT, which demonstrated a hyperdense pelvic lesion. The drain was retrieved via laparotomy, with immediate resolution of the patient’s chronic abdominal pain [8]. Dzefi-Tettey et al. reported a retained pelvic drain tube identified incidentally on ultrasonography performed for an unrelated indication in an otherwise asymptomatic patient, noting that recognition required a high index of suspicion on the part of the reporting radiologist [9]. Collectively, these reports establish that retained drains need not produce overt symptoms, and that their discovery is not reliably predictable on clinical grounds alone.

The present case extends this literature in several respects. The retained fragment was discovered incidentally during elective colostomy reversal, underscoring that vigilance is warranted even during planned, non-emergency re-operative surgery. More strikingly, the drain had become functionally incorporated into surrounding structures, its mid-segment forming a fibrous tract that sealed a 2 × 1 cm ileal perforation, its proximal end eroding into the jejunum, and its transected distal end embedded within the anterior abdominal wall, an anatomical configuration in which the foreign body appeared to act paradoxically as a biological seal, plausibly explaining the patient’s prolonged asymptomatic course (Figure 2). The retained fragment was located on the left side of the abdomen, corresponding to the site of the pelvic drain placed at the index laparotomy, which is brought out through the left abdominal wall, as distinct from the subhepatic drain, sited on the right. Rather than invoking substantial migration, the fragment’s position at retrieval most likely reflects the original course of the pelvic drain, with progressive fibrous encasement and adhesion formation fixing it to adjacent structures in situ over the 10-month interval.

As noted, the available operative records did not confirm that the drain had been removed intact; this reflects a critical gap not in the availability of documentation, but in its content, and distinguishes this case from prior reports in which retained fragments typically went undocumented at any stage of the patient’s care. The segment of drain bridging the ileal perforation was fenestrated, raising the possibility that luminal content could drain internally through these fenestrations along the established fibrous tract rather than spilling freely into the peritoneum, a mechanism that would account for the absence of contamination regardless of whether the perforation arose from chronic pressure erosion by the drain or represented an unrecognized enterotomy at the index surgery that was fortuitously sealed by the adjacent drain from the outset. As the specimen was not submitted for histopathological examination and drain output from the index admission was not documented, these two mechanisms cannot be definitively distinguished on the available evidence. The prolonged asymptomatic course in this patient may itself have been contingent on fecal diversion: the de-functioning colostomy limited luminal flow across the sealed ileal defect, and the reported literature suggests such fragments tend to declare themselves eventually, whether through delayed symptoms or incidental detection. Had the patient not been diverted, or had earlier reoperation disturbed the sealed configuration, the indolent course observed here might not have persisted.

A further observation concerns preoperative imaging. ADK drains are not radio-opaque on conventional radiography unless a radio-opaque marker is present, and in the present case preoperative ultrasonography was reported as normal; however, it had been performed as routine preoperative assessment rather than with any suspicion of a retained foreign body. This is consistent with the observation that ultrasound detection of tubular foreign bodies embedded within adhesive fibrous tissue depends heavily on the radiologist’s index of suspicion rather than on the inherent capability of the modality, as demonstrated by Dzefi-Tettey et al., in whom such a drain was identified, albeit only incidentally, on ultrasonography performed for an unrelated indication [8-10]. This mirrors the diagnostic pathway described by Irpatgire et al., in whom ultrasonography was repeatedly unrevealing, and CT proved diagnostic [8]. Imaging pitfalls of this nature are not unique to drains; retained or residual material of various kinds has similarly been reported to mimic or evade detection on standard imaging [11]. Accordingly, cross-sectional imaging should be considered as part of the preoperative workup in any patient presenting for re-operative abdominal surgery whose prior operative records do not explicitly confirm that all drains were removed intact, even where documentation is otherwise complete, and drain removal itself is recorded.

More broadly, this case reinforces that documentation of drain removal must extend beyond confirming that a drain was removed to explicitly recording that it was removed intact, a distinction not captured by standard item-counting protocols [12].

## Conclusions

The present case documents a rare intraoperative finding of a retained ADK drain fragment, the consequence of incomplete drain removal with unrecognized transection, that had undergone fibrous incorporation into adjacent small bowel, sealing an ileal perforation and sustaining a clinically occult configuration until its incidental discovery during elective colostomy reversal. This case illustrates that a retained surgical item may remain asymptomatic for a prolonged interval, particularly where factors such as fecal diversion limit the consequences of the underlying defect, and that symptomatic quiescence should not be taken as evidence of its absence. It further highlights the value of confirming that a drain has been removed intact, with visual inspection of the removed drain, rather than simply recording that it was removed, and of reviewing prior operative records before abdominal re-entry. Heightened awareness of this possibility may aid in the recognition and prevention of similar occurrences.
